# Plasma biomarkers predict incident cognitive decline up to 29 years prior to disease onset: a memory clinic cohort study of 4,073 participants

**DOI:** 10.1002/alz70856_106702

**Published:** 2026-01-14

**Authors:** Xuemei Zeng, Rebecca A Deek, Michel N Nafash, Jeremy M. Gu, Lamia Choity, Tara K Lafferty, Marissa F Farinas, Margaret A Bedison, Rocco B Mercurio, Cristy Matan, Alexandra Gogola, Julia K. Kofler, Dana L Tudorascu, C. Elizabeth Shaaban, Jennifer H Lingler, Tharick A Pascoal, William E Klunk, Victor L. Villemagne, Milos D. Ikonomovic, Sarah B Berman, Robert Sweet, Beth E. Snitz, Ann D Cohen, M. Ilyas Kamboh, Oscar L Lopez, Thomas K Karikari

**Affiliations:** ^1^ University of Pittsburgh School of Medicine, Pittsburgh, PA, USA; ^2^ University of Pittsburgh, Pittsburgh, PA, USA; ^3^ School of Public Health, University of Pittsburgh, Pittsburgh, PA, USA; ^4^ University of Pittsburgh Alzheimer's Disease Research Center (ADRC), Pittsburgh, PA, USA; ^5^ University of Pittsburgh, School of Medicine, Pittsburgh, PA, USA

## Abstract

**Background:**

Plasma biomarkers have demonstrated excellent performances in detecting AD/ADRD‐related brain pathology. However, their relationship with cognitive decline remains unclear. We examined this in a large memory clinic cohort with baseline plasma biomarkers and repeated cognitive assessments over approximately three decades.

**Method:**

Participants at the University of Pittsburgh Alzheimer's Disease Research Center underwent blood collection and Clinical Dementia Rating (CDR) Sum of Boxes‐based cognitive assessment cross‐sectionally, followed by annual CDR assessments for up to 29 years (3.0 [IQR 1.9‐5.9]). Plasma *p*‐tau181, *p*‐tau217, brain‐derived tau (BD‐tau), GFAP and NfL, were measured using SIMOA assays. Linear/logistic regression and Fisher's exact were employed for statistical inference.

**Result:**

We included 4,073 participants (59.9% female; 90.2% self‐identified non‐Hispanic White), aged 71.9 ± 9.8 years, with 2160 being non‐demented (CDR≤0.5) at baseline. Cross‐sectionally, higher levels of all biomarkers were significantly associated with worse CDR scores. Longitudinally, baseline *p*‐tau181 and GFAP levels best predicted cognitive decline at 0‐2, 2‐5, 5‐10, and >10 years. In contrast, *p*‐tau217 was superior at predicting whether cognitive decline would happen at all within 2, 5, or 10 years, with AUCs up to 0.810. Participants with above‐median *p*‐tau217 levels had the highest odds of cognitive decline (2.57, 4.53, and 10.34 times within 2, 5 and 10 years, respectively). *p*‐tau217, and *p*‐tau217/BD‐tau ratio accounting for CNS‐derived *p*‐tau217, were most effective in predicting cognitive decline in participants who were cognitively non‐demented at baseline. Importantly, cognitively stable individuals had lower levels of all plasma biomarkers vs. progressors, with *p*‐tau217 best at separating these groups.

**Conclusion:**

Leveraging a large cohort with extensive longitudinal data, our findings underscore the significant value of blood‐based biomarkers in predicting cognitive decline to aid personalized clinical management and ultimately improve patient outcomes.

